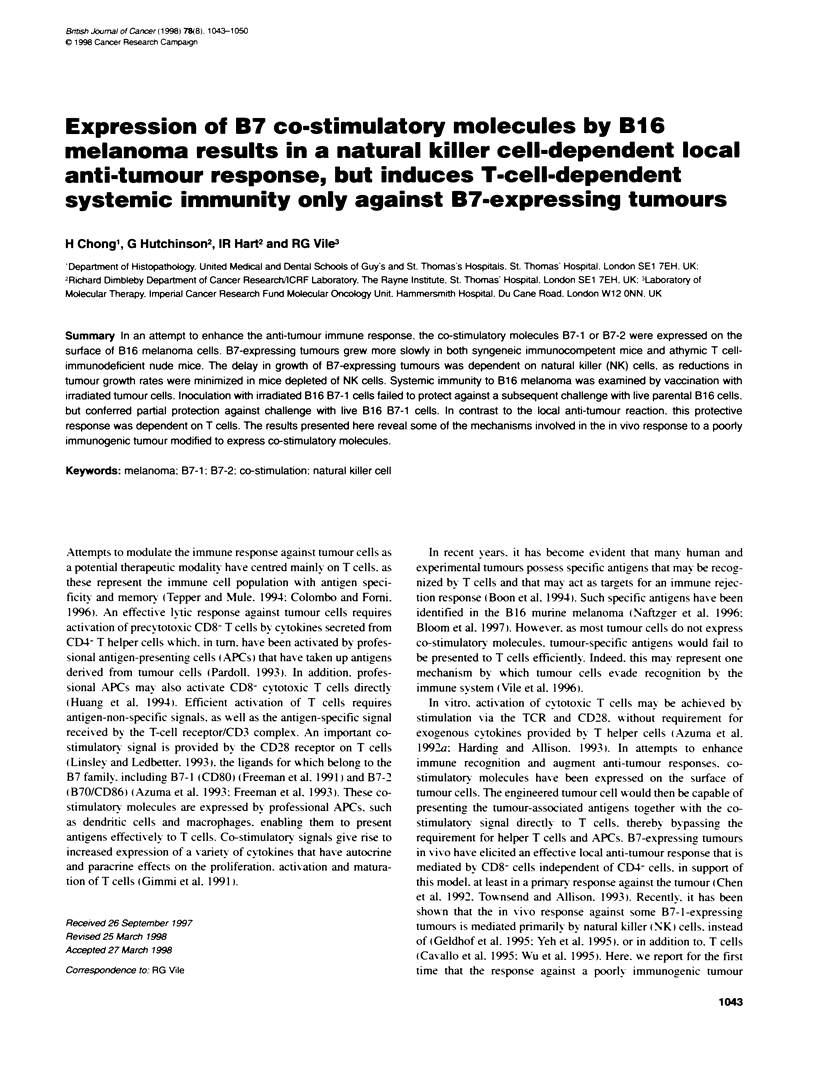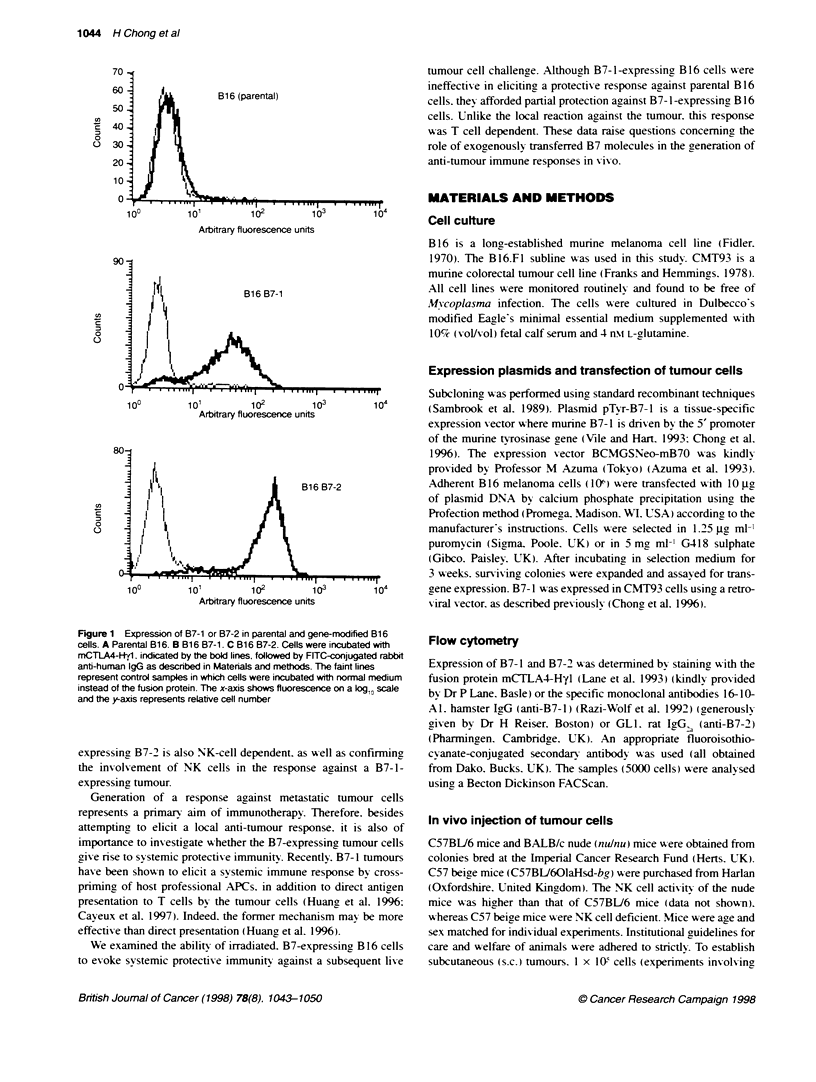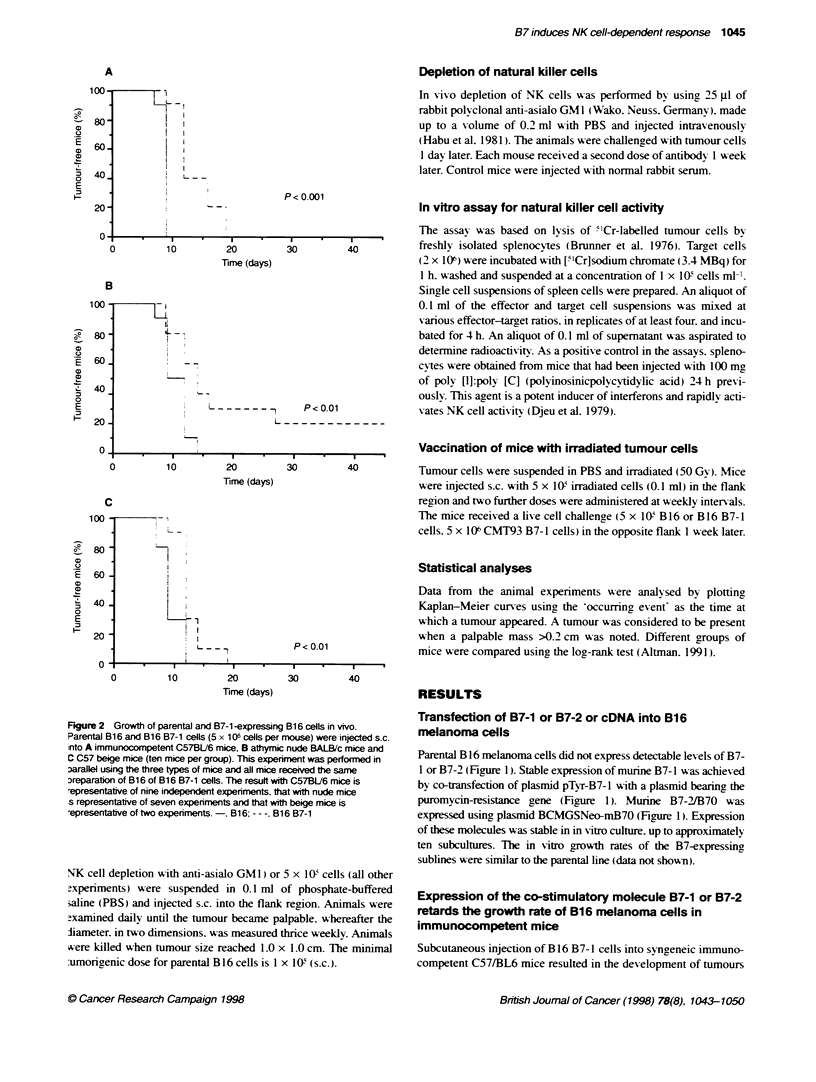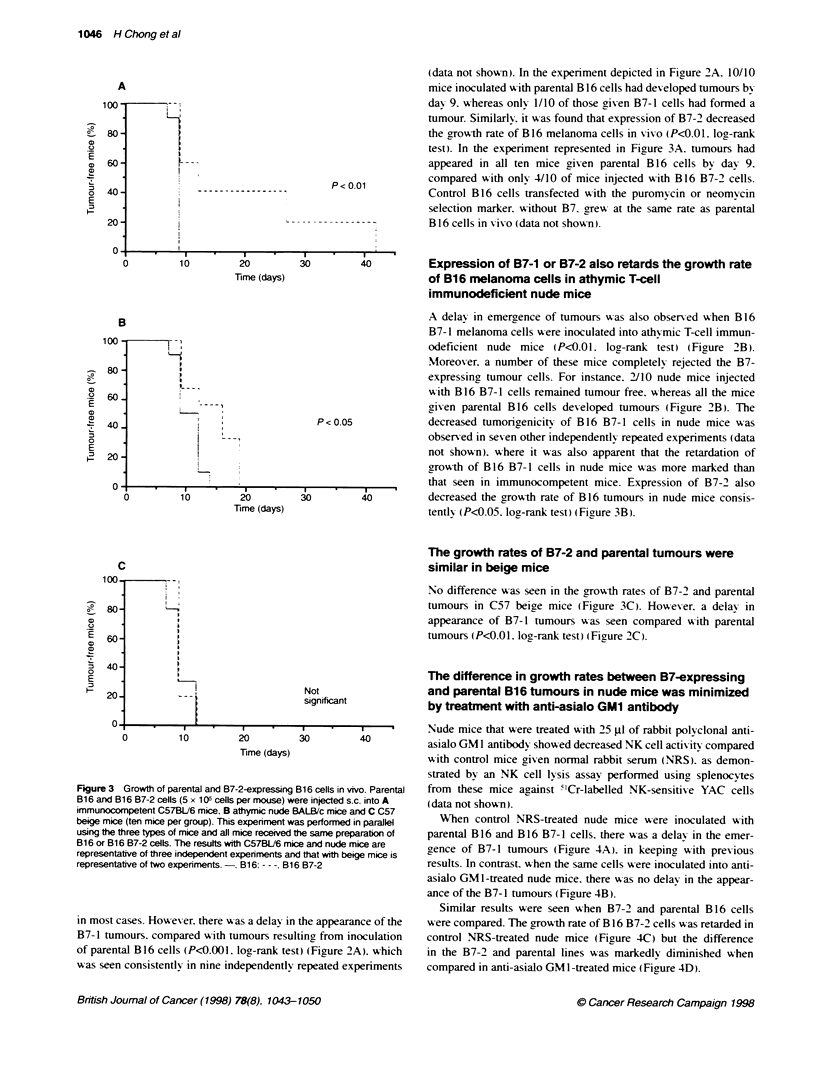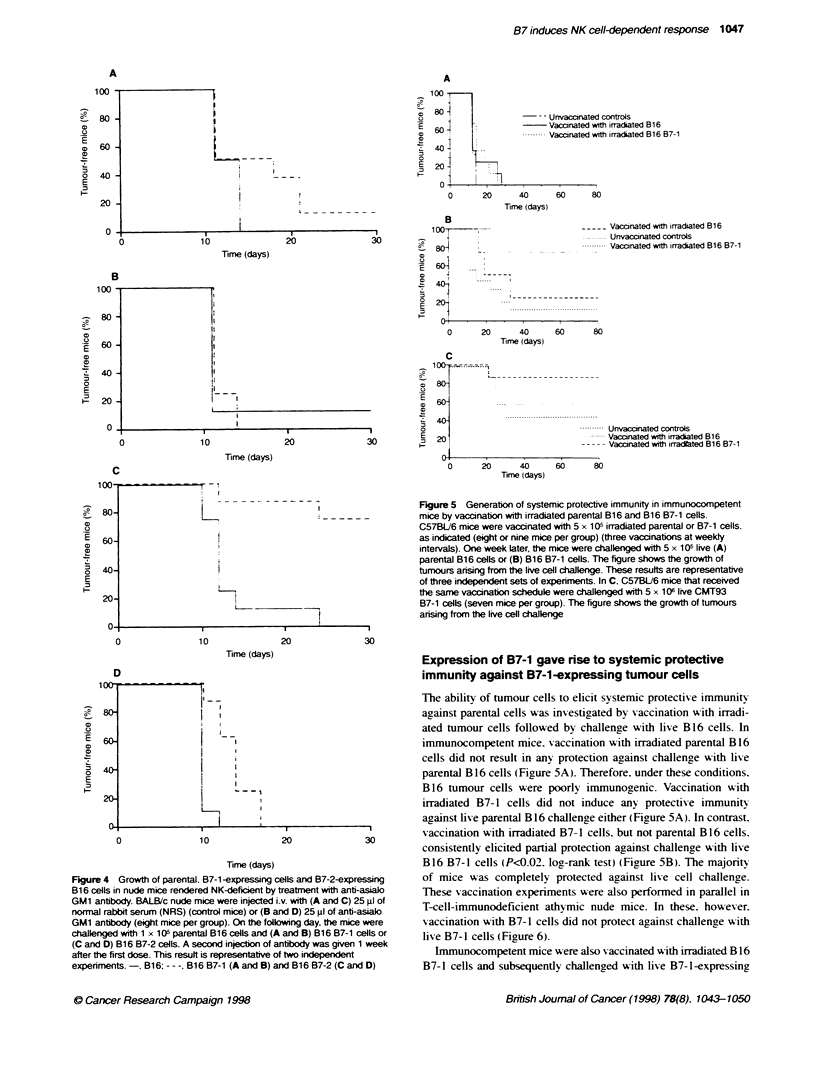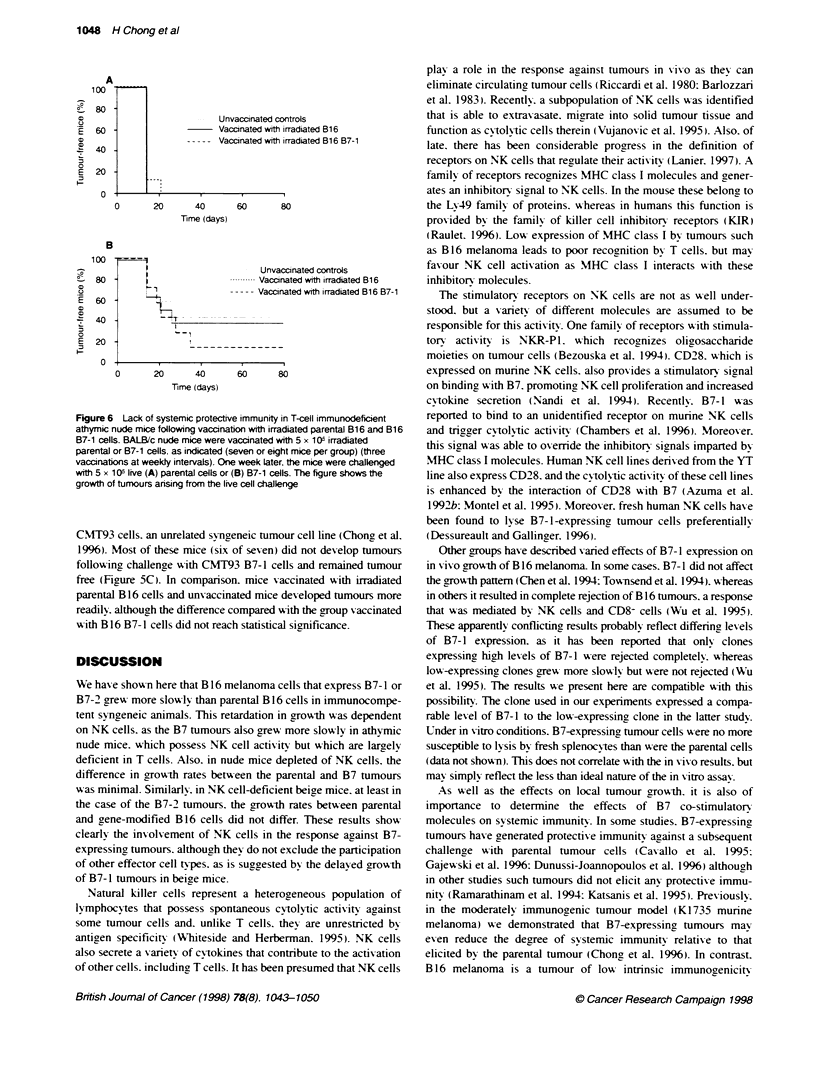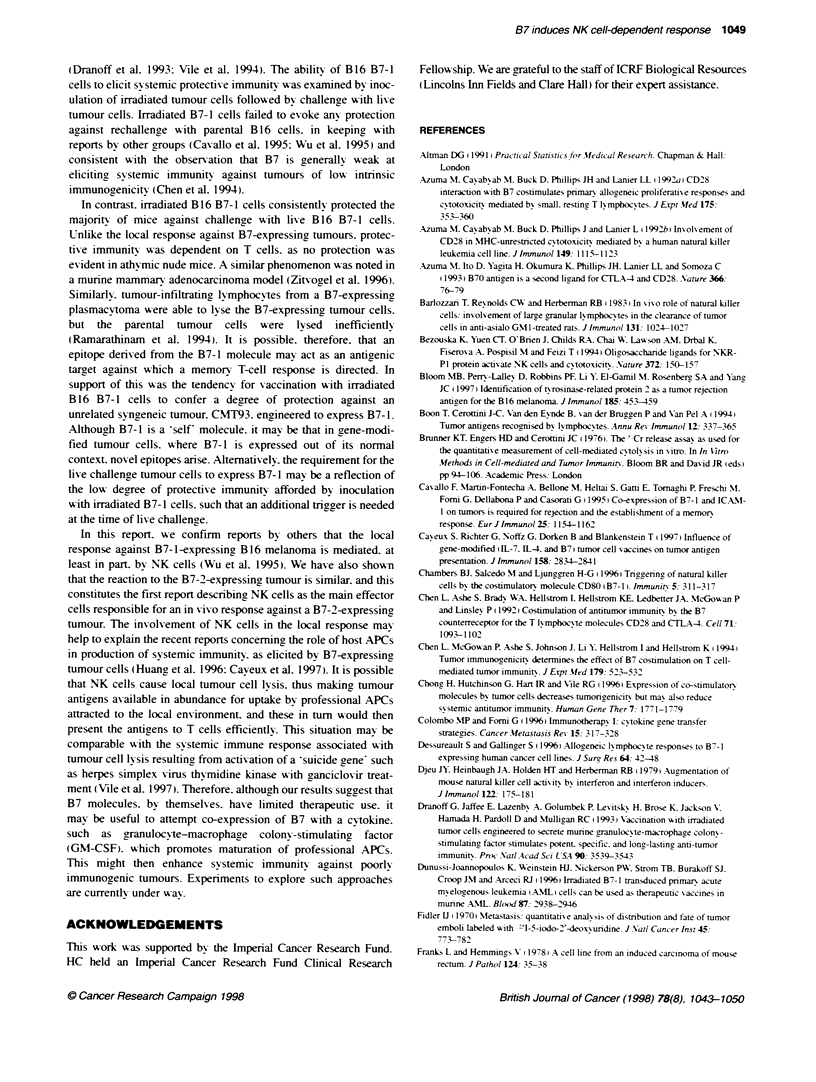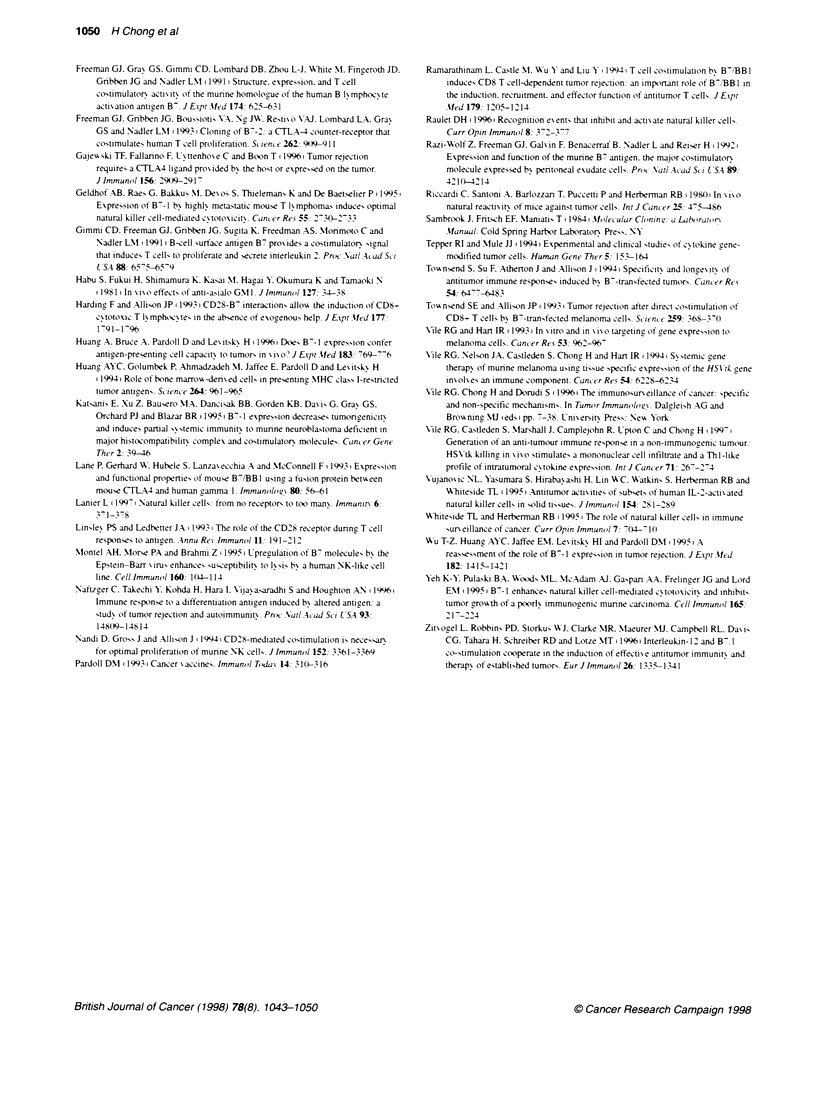# Expression of B7 co-stimulatory molecules by B16 melanoma results in a natural killer cell-dependent local anti-tumour response, but induces T-cell-dependent systemic immunity only against B7-expressing tumours.

**DOI:** 10.1038/bjc.1998.625

**Published:** 1998-10

**Authors:** H. Chong, G. Hutchinson, I. R. Hart, R. G. Vile

**Affiliations:** Department of Histopathology, United Medical and Dental Schools of Guy's and St. Thomas's Hospitals, London, UK.

## Abstract

In an attempt to enhance the anti-tumour immune response, the co-stimulatory molecules B7-1 or B7-2 were expressed on the surface of B16 melanoma cells. B7-expressing tumours grew more slowly in both syngeneic immunocompetent mice and athymic T cell-immunodeficient nude mice. The delay in growth of B7-expressing tumours was dependent on natural killer (NK) cells, as reductions in tumour growth rates were minimized in mice depleted of NK cells. Systemic immunity to B16 melanoma was examined by vaccination with irradiated tumour cells. Inoculation with irradiated B16 B7-1 cells failed to protect against a subsequent challenge with live parental B16 cells, but conferred partial protection against challenge with live B16 B7-1 cells. In contrast to the local anti-tumour reaction, this protective response was dependent on T cells. The results presented here reveal some of the mechanisms involved in the in vivo response to a poorly immunogenic tumour modified to express co-stimulatory molecules.


British Journal of Cancer (1998) 78(8), 1043–1050
© 1998 Cancer Research Campaign

### Expression of B7 co-stimulatory molecules by B16 melanoma results in a natural killer cell-dependent local anti-tumour response, but induces T-cell-dependent systemic immunity only against B7-expressing tumours

**H Chong[1], G Hutchinson[2], IR Hart[2] and RG Vile[3]**

[1]Department of Histopathology, United Medical and Dental Schools of Guy's and St. Thomas's Hospitals, St. Thomas' Hospital, London SE1 7EH, UK; [2]Richard Dimbleby Department of Cancer Research/ICRF Laboratory, The Rayne Institute, St. Thomas' Hospital, London SE1 7EH, UK; [3]Laboratory of Molecular Therapy, Imperial Cancer Research Fund Molecular Oncology Unit, Hammersmith Hospital, Du Cane Road, London W12 0NN, UK

**Summary** In an attempt to enhance the anti-tumour immune response, the co-stimulatory molecules B7-1 or B7-2 were expressed on the surface of B16 melanoma cells. B7-expressing tumours grew more slowly in both syngeneic immunocompetent mice and athymic T cell-immunodeficient nude mice. The delay in growth of B7-expressing tumours was dependent on natural killer (NK) cells, as reductions in tumour growth rates were minimized in mice depleted of NK cells. Systemic immunity to B16 melanoma was examined by vaccination with irradiated tumour cells. Inoculation with irradiated B16 B7-1 cells failed to protect against a subsequent challenge with live parental B16 cells, but conferred partial protection against challenge with live B16 B7-1 cells. In contrast to the local anti-tumour reaction, this protective response was dependent on T cells. The results presented here reveal some of the mechanisms involved in the in vivo response to a poorly immunogenic tumour modified to express co-stimulatory molecules.

**Keywords:** melanoma; B7-1; B7-2; co-stimulation; natural killer cell

Attempts to modulate the immune response against tumour cells as a potential therapeutic modality have centred mainly on T cells, as these represent the immune cell population with antigen specificity and memory (Tepper and Mule, 1994; Colombo and Forni, 1996). An effective lytic response against tumour cells requires activation of precytotoxic CD8$^+$ T cells by cytokines secreted from CD4$^+$ T helper cells which, in turn, have been activated by professional antigen-presenting cells (APCs) that have taken up antigens derived from tumour cells (Pardoll, 1993). In addition, professional APCs may also activate CD8$^+$ cytotoxic T cells directly (Huang et al, 1994). Efficient activation of T cells requires antigen-non-specific signals, as well as the antigen-specific signal received by the T-cell receptor/CD3 complex. An important co-stimulatory signal is provided by the CD28 receptor on T cells (Linsley and Ledbetter, 1993), the ligands for which belong to the B7 family, including B7-1 (CD80) (Freeman et al, 1991) and B7-2 (B70/CD86) (Azuma et al, 1993; Freeman et al, 1993). These co-stimulatory molecules are expressed by professional APCs, such as dendritic cells and macrophages, enabling them to present antigens effectively to T cells. Co-stimulatory signals give rise to increased expression of a variety of cytokines that have autocrine and paracrine effects on the proliferation, activation and maturation of T cells (Gimmi et al, 1991).

*Received 26 September 1997*
*Revised 25 March 1998*
*Accepted 27 March 1998*

*Correspondence to:* RG Vile

In recent years, it has become evident that many human and experimental tumours possess specific antigens that may be recognized by T cells and that may act as targets for an immune rejection response (Boon et al, 1994). Such specific antigens have been identified in the B16 murine melanoma (Naftzger et al, 1996; Bloom et al, 1997). However, as most tumour cells do not express co-stimulatory molecules, tumour-specific antigens would fail to be presented to T cells efficiently. Indeed, this may represent one mechanism by which tumour cells evade recognition by the immune system (Vile et al, 1996).

In vitro, activation of cytotoxic T cells may be achieved by stimulation via the TCR and CD28, without requirement for exogenous cytokines provided by T helper cells (Azuma et al, 1992*a*; Harding and Allison, 1993). In attempts to enhance immune recognition and augment anti-tumour responses, co-stimulatory molecules have been expressed on the surface of tumour cells. The engineered tumour cell would then be capable of presenting the tumour-associated antigens together with the co-stimulatory signal directly to T cells, thereby bypassing the requirement for helper T cells and APCs. B7-expressing tumours in vivo have elicited an effective local anti-tumour response that is mediated by CD8$^+$ cells independent of CD4$^+$ cells, in support of this model, at least in a primary response against the tumour (Chen et al, 1992, Townsend and Allison, 1993). Recently, it has been shown that the in vivo response against some B7-1-expressing tumours is mediated primarily by natural killer (NK) cells, instead of (Geldhof et al, 1995; Yeh et al, 1995), or in addition to, T cells (Cavallo et al, 1995; Wu et al, 1995). Here, we report for the first time that the response against a poorly immunogenic tumour

**Figure 1** Expression of B7-1 or B7-2 in parental and gene-modified B16 cells. **A** Parental B16. **B** B16 B7-1. **C** B16 B7-2. Cells were incubated with mCTLA4-Hγ1, indicated by the bold lines, followed by FITC-conjugated rabbit anti-human IgG as described in Materials and methods. The faint lines represent control samples in which cells were incubated with normal medium instead of the fusion protein. The *x*-axis shows fluorescence on a $\log_{10}$ scale and the *y*-axis represents relative cell number expressing B7-2 is also NK-cell dependent, as well as confirming the involvement of NK cells in the response against a B7-1-expressing tumour.

Generation of a response against metastatic tumour cells represents a primary aim of immunotherapy. Therefore, besides attempting to elicit a local anti-tumour response, it is also of importance to investigate whether the B7-expressing tumour cells give rise to systemic protective immunity. Recently, B7-1 tumours have been shown to elicit a systemic immune response by cross-priming of host professional APCs, in addition to direct antigen presentation to T cells by the tumour cells (Huang et al, 1996; Cayeux et al, 1997). Indeed, the former mechanism may be more effective than direct presentation (Huang et al, 1996).

We examined the ability of irradiated, B7-expressing B16 cells to evoke systemic protective immunity against a subsequent live tumour cell challenge. Although B7-1-expressing B16 cells were ineffective in eliciting a protective response against parental B16 cells, they afforded partial protection against B7-1-expressing B16 cells. Unlike the local reaction against the tumour, this response was T cell dependent. These data raise questions concerning the role of exogenously transferred B7 molecules in the generation of anti-tumour immune responses in vivo.

### MATERIALS AND METHODS

### Cell culture

B16 is a long-established murine melanoma cell line (Fidler, 1970). The B16.F1 subline was used in this study. CMT93 is a murine colorectal tumour cell line (Franks and Hemmings, 1978). All cell lines were monitored routinely and found to be free of *Mycoplasma* infection. The cells were cultured in Dulbecco's modified Eagle's minimal essential medium supplemented with 10% (vol/vol) fetal calf serum and 4 nM L-glutamine.

### Expression plasmids and transfection of tumour cells

Subcloning was performed using standard recombinant techniques (Sambrook et al, 1989). Plasmid pTyr-B7-1 is a tissue-specific expression vector where murine B7-1 is driven by the 5′ promoter of the murine tyrosinase gene (Vile and Hart, 1993; Chong et al, 1996). The expression vector BCMGSNeo-mB70 was kindly provided by Professor M Azuma (Tokyo) (Azuma et al, 1993). Adherent B16 melanoma cells ($10^6$) were transfected with 10 μg of plasmid DNA by calcium phosphate precipitation using the Profection method (Promega, Madison, WI, USA) according to the manufacturer's instructions. Cells were selected in 1.25 μg $ml^{-1}$ puromycin (Sigma, Poole, UK) or in 5 mg $ml^{-1}$ G418 sulphate (Gibco, Paisley, UK). After incubating in selection medium for 3 weeks, surviving colonies were expanded and assayed for transgene expression. B7-1 was expressed in CMT93 cells using a retroviral vector, as described previously (Chong et al, 1996).

### Flow cytometry

Expression of B7-1 and B7-2 was determined by staining with the fusion protein mCTLA4-Hγ1 (Lane et al, 1993) (kindly provided by Dr P Lane, Basle) or the specific monoclonal antibodies 16-10-A1, hamster IgG (anti-B7-1) (Razi-Wolf et al, 1992) (generously given by Dr H Reiser, Boston) or GL1, rat $IgG_{2a}$ (anti-B7-2) (Pharmingen, Cambridge, UK). An appropriate fluoroisothiocyanate-conjugated secondary antibody was used (all obtained from Dako, Bucks, UK). The samples (5000 cells) were analysed using a Becton Dickinson FACScan.

### In vivo injection of tumour cells

C57BL/6 mice and BALB/c nude (*nu/nu*) mice were obtained from colonies bred at the Imperial Cancer Research Fund (Herts, UK). C57 beige mice (C57BL/6OlaHsd-*bg*) were purchased from Harlan (Oxfordshire, United Kingdom). The NK cell activity of the nude mice was higher than that of C57BL/6 mice (data not shown), whereas C57 beige mice were NK cell deficient. Mice were age and sex matched for individual experiments. Institutional guidelines for care and welfare of animals were adhered to strictly. To establish subcutaneous (s.c.) tumours, 1 × $10^5$ cells (experiments involving

 © Cancer Research Campaign 1998

**Figure 2** Growth of parental and B7-1-expressing B16 cells in vivo. Parental B16 and B16 B7-1 cells ($5 \times 10^5$ cells per mouse) were injected s.c. into **A** immunocompetent C57BL/6 mice, **B** athymic nude BALB/c mice and **C** C57 beige mice (ten mice per group). This experiment was performed in parallel using the three types of mice and all mice received the same preparation of B16 of B16 B7-1 cells. The result with C57BL/6 mice is representative of nine independent experiments, that with nude mice is representative of seven experiments and that with beige mice is representative of two experiments. —, B16; - - -, B16 B7-1

NK cell depletion with anti-asialo GM1) or $5 \times 10^5$ cells (all other experiments) were suspended in 0.1 ml of phosphate-buffered saline (PBS) and injected s.c. into the flank region. Animals were examined daily until the tumour became palpable, whereafter the diameter, in two dimensions, was measured thrice weekly. Animals were killed when tumour size reached $1.0 \times 1.0$ cm. The minimal tumorigenic dose for parental B16 cells is $1 \times 10^5$ (s.c.).

### Depletion of natural killer cells

In vivo depletion of NK cells was performed by using 25 µl of rabbit polyclonal anti-asialo GM1 (Wako, Neuss, Germany), made up to a volume of 0.2 ml with PBS and injected intravenously (Habu et al, 1981). The animals were challenged with tumour cells 1 day later. Each mouse received a second dose of antibody 1 week later. Control mice were injected with normal rabbit serum.

### In vitro assay for natural killer cell activity

The assay was based on lysis of $^{51}$Cr-labelled tumour cells by freshly isolated splenocytes (Brunner et al, 1976). Target cells ($2 \times 10^6$) were incubated with [$^{51}$Cr]sodium chromate (3.4 MBq) for 1 h, washed and suspended at a concentration of $1 \times 10^5$ cells ml$^{-1}$. Single cell suspensions of spleen cells were prepared. An aliquot of 0.1 ml of the effector and target cell suspensions was mixed at various effector–target ratios, in replicates of at least four, and incubated for 4 h. An aliquot of 0.1 ml of supernatant was aspirated to determine radioactivity. As a positive control in the assays, splenocytes were obtained from mice that had been injected with 100 mg of poly [I]:poly [C] (polyinosinicpolycytidylic acid) 24 h previously. This agent is a potent inducer of interferons and rapidly activates NK cell activity (Djeu et al, 1979).

### Vaccination of mice with irradiated tumour cells

Tumour cells were suspended in PBS and irradiated (50 Gy). Mice were injected s.c. with $5 \times 10^5$ irradiated cells (0.1 ml) in the flank region and two further doses were administered at weekly intervals. The mice received a live cell challenge ($5 \times 10^5$ B16 or B16 B7-1 cells, $5 \times 10^6$ CMT93 B7-1 cells) in the opposite flank 1 week later.

### Statistical analyses

Data from the animal experiments were analysed by plotting Kaplan–Meier curves using the 'occurring event' as the time at which a tumour appeared. A tumour was considered to be present when a palpable mass >0.2 cm was noted. Different groups of mice were compared using the log-rank test (Altman, 1991).

### RESULTS

### Transfection of B7-1 or B7-2 or cDNA into B16 melanoma cells

Parental B16 melanoma cells did not express detectable levels of B7-1 or B7-2 (Figure 1). Stable expression of murine B7-1 was achieved by co-transfection of plasmid pTyr-B7-1 with a plasmid bearing the puromycin-resistance gene (Figure 1). Murine B7-2/B70 was expressed using plasmid BCMGSNeo-mB70 (Figure 1). Expression of these molecules was stable in in vitro culture, up to approximately ten subcultures. The in vitro growth rates of the B7-expressing sublines were similar to the parental line (data not shown).

### Expression of the co-stimulatory molecule B7-1 or B7-2 retards the growth rate of B16 melanoma cells in immunocompetent mice

Subcutaneous injection of B16 B7-1 cells into syngeneic immunocompetent C57/BL6 mice resulted in the development of tumours

© Cancer Research Campaign 1998

**Figure 3** Growth of parental and B7-2-expressing B16 cells in vivo. Parental B16 and B16 B7-2 cells ($5 \times 10^5$ cells per mouse) were injected s.c. into **A** immunocompetent C57BL/6 mice, **B** athymic nude BALB/c mice and **C** C57 beige mice (ten mice per group). This experiment was performed in parallel using the three types of mice and all mice received the same preparation of B16 or B16 B7-2 cells. The results with C57BL/6 mice and nude mice are representative of three independent experiments and that with beige mice is representative of two experiments. —, B16; - - -, B16 B7-2

in most cases. However, there was a delay in the appearance of the B7-1 tumours, compared with tumours resulting from inoculation of parental B16 cells (*P*<0.001, log-rank test) (Figure 2A), which was seen consistently in nine independently repeated experiments (data not shown). In the experiment depicted in Figure 2A, 10/10 mice inoculated with parental B16 cells had developed tumours by day 9, whereas only 1/10 of those given B7-1 cells had formed a tumour. Similarly, it was found that expression of B7-2 decreased the growth rate of B16 melanoma cells in vivo (*P*<0.01, log-rank test). In the experiment represented in Figure 3A, tumours had appeared in all ten mice given parental B16 cells by day 9, compared with only 4/10 of mice injected with B16 B7-2 cells. Control B16 cells transfected with the puromycin or neomycin selection marker, without B7, grew at the same rate as parental B16 cells in vivo (data not shown).

### Expression of B7-1 or B7-2 also retards the growth rate of B16 melanoma cells in athymic T-cell immunodeficient nude mice

A delay in emergence of tumours was also observed when B16 B7-1 melanoma cells were inoculated into athymic T-cell immunodeficient nude mice (*P*<0.01, log-rank test) (Figure 2B). Moreover, a number of these mice completely rejected the B7-expressing tumour cells. For instance, 2/10 nude mice injected with B16 B7-1 cells remained tumour free, whereas all the mice given parental B16 cells developed tumours (Figure 2B). The decreased tumorigenicity of B16 B7-1 cells in nude mice was observed in seven other independently repeated experiments (data not shown), where it was also apparent that the retardation of growth of B16 B7-1 cells in nude mice was more marked than that seen in immunocompetent mice. Expression of B7-2 also decreased the growth rate of B16 tumours in nude mice consistently (*P*<0.05, log-rank test) (Figure 3B).

### The growth rates of B7-2 and parental tumours were similar in beige mice

No difference was seen in the growth rates of B7-2 and parental tumours in C57 beige mice (Figure 3C). However, a delay in appearance of B7-1 tumours was seen compared with parental tumours (*P*<0.01, log-rank test) (Figure 2C).

### The difference in growth rates between B7-expressing and parental B16 tumours in nude mice was minimized by treatment with anti-asialo GM1 antibody

Nude mice that were treated with 25 µl of rabbit polyclonal anti-asialo GM1 antibody showed decreased NK cell activity compared with control mice given normal rabbit serum (NRS), as demonstrated by an NK cell lysis assay performed using splenocytes from these mice against $^{51}$Cr-labelled NK-sensitive YAC cells (data not shown).

When control NRS-treated nude mice were inoculated with parental B16 and B16 B7-1 cells, there was a delay in the emergence of B7-1 tumours (Figure 4A), in keeping with previous results. In contrast, when the same cells were inoculated into anti-asialo GM1-treated nude mice, there was no delay in the appearance of the B7-1 tumours (Figure 4B).

Similar results were seen when B7-2 and parental B16 cells were compared. The growth rate of B16 B7-2 cells was retarded in control NRS-treated nude mice (Figure 4C) but the difference in the B7-2 and parental lines was markedly diminished when compared in anti-asialo GM1-treated mice (Figure 4D).

© Cancer Research Campaign 1998

**Figure 4** Growth of parental, B7-1-expressing cells and B7-2-expressing B16 cells in nude mice rendered NK-deficient by treatment with anti-asialo GM1 antibody. BALB/c nude mice were injected i.v. with (**A** and **C**) 25 µl of normal rabbit serum (NRS) (control mice) or (**B** and **D**) 25 µl of anti-asialo GM1 antibody (eight mice per group). On the following day, the mice were challenged with 1 × $10^5$ parental B16 cells and (**A** and **B**) B16 B7-1 cells or (**C** and **D**) B16 B7-2 cells. A second injection of antibody was given 1 week after the first dose. This result is representative of two independent experiments. —, B16; - - -, B16 B7-1 (**A** and **B**) and B16 B7-2 (**C** and **D**)

**Figure 5** Generation of systemic protective immunity in immunocompetent mice by vaccination with irradiated parental B16 and B16 B7-1 cells. C57BL/6 mice were vaccinated with 5 × $10^5$ irradiated parental or B7-1 cells, as indicated (eight or nine mice per group) (three vaccinations at weekly intervals). One week later, the mice were challenged with 5 × $10^5$ live (**A**) parental B16 cells or (**B**) B16 B7-1 cells. The figure shows the growth of tumours arising from the live cell challenge. These results are representative of three independent sets of experiments. In **C**, C57BL/6 mice that received the same vaccination schedule were challenged with 5 × $10^6$ live CMT93 B7-1 cells (seven mice per group). The figure shows the growth of tumours arising from the live cell challenge

### Expression of B7-1 gave rise to systemic protective immunity against B7-1-expressing tumour cells

The ability of tumour cells to elicit systemic protective immunity against parental cells was investigated by vaccination with irradiated tumour cells followed by challenge with live B16 cells. In immunocompetent mice, vaccination with irradiated parental B16 cells did not result in any protection against challenge with live parental B16 cells (Figure 5A). Therefore, under these conditions, B16 tumour cells were poorly immunogenic. Vaccination with irradiated B7-1 cells did not induce any protective immunity against live parental B16 challenge either (Figure 5A). In contrast, vaccination with irradiated B7-1 cells, but not parental B16 cells, consistently elicited partial protection against challenge with live B16 B7-1 cells ($P<0.02$, log-rank test) (Figure 5B). The majority of mice was completely protected against live cell challenge. These vaccination experiments were also performed in parallel in T-cell-immunodeficient athymic nude mice. In these, however, vaccination with B7-1 cells did not protect against challenge with live B7-1 cells (Figure 6).

Immunocompetent mice were also vaccinated with irradiated B16 B7-1 cells and subsequently challenged with live B7-1-expressing

**Figure 6** Lack of systemic protective immunity in T-cell immunodeficient athymic nude mice following vaccination with irradiated parental B16 and B16 B7-1 cells. BALB/c nude mice were vaccinated with 5 × $10^5$ irradiated parental or B7-1 cells, as indicated (seven or eight mice per group) (three vaccinations at weekly intervals). One week later, the mice were challenged with 5 × $10^5$ live (**A**) parental cells or (**B**) B7-1 cells. The figure shows the growth of tumours arising from the live cell challenge

CMT93 cells, an unrelated syngeneic tumour cell line (Chong et al, 1996). Most of these mice (six of seven) did not develop tumours following challenge with CMT93 B7-1 cells and remained tumour free (Figure 5C). In comparison, mice vaccinated with irradiated parental B16 cells and unvaccinated mice developed tumours more readily, although the difference compared with the group vaccinated with B16 B7-1 cells did not reach statistical significance.

### DISCUSSION

We have shown here that B16 melanoma cells that express B7-1 or B7-2 grew more slowly than parental B16 cells in immunocompetent syngeneic animals. This retardation in growth was dependent on NK cells, as the B7 tumours also grew more slowly in athymic nude mice, which possess NK cell activity but which are largely deficient in T cells. Also, in nude mice depleted of NK cells, the difference in growth rates between the parental and B7 tumours was minimal. Similarly, in NK cell-deficient beige mice, at least in the case of the B7-2 tumours, the growth rates between parental and gene-modified B16 cells did not differ. These results show clearly the involvement of NK cells in the response against B7-expressing tumours, although they do not exclude the participation of other effector cell types, as is suggested by the delayed growth of B7-1 tumours in beige mice.

Natural killer cells represent a heterogeneous population of lymphocytes that possess spontaneous cytolytic activity against some tumour cells and, unlike T cells, they are unrestricted by antigen specificity (Whiteside and Herberman, 1995). NK cells also secrete a variety of cytokines that contribute to the activation of other cells, including T cells. It has been presumed that NK cells play a role in the response against tumours in vivo as they can eliminate circulating tumour cells (Riccardi et al, 1980; Barlozzari et al, 1983). Recently, a subpopulation of NK cells was identified that is able to extravasate, migrate into solid tumour tissue and function as cytolytic cells therein (Vujanovic et al, 1995). Also, of late, there has been considerable progress in the definition of receptors on NK cells that regulate their activity (Lanier, 1997). A family of receptors recognizes MHC class I molecules and generates an inhibitory signal to NK cells. In the mouse these belong to the Ly49 family of proteins, whereas in humans this function is provided by the family of killer cell inhibitory receptors (KIR) (Raulet, 1996). Low expression of MHC class I by tumours such as B16 melanoma leads to poor recognition by T cells, but may favour NK cell activation as MHC class I interacts with these inhibitory molecules.

The stimulatory receptors on NK cells are not as well understood, but a variety of different molecules are assumed to be responsible for this activity. One family of receptors with stimulatory activity is NKR-P1, which recognizes oligosaccharide moieties on tumour cells (Bezouska et al, 1994). CD28, which is expressed on murine NK cells, also provides a stimulatory signal on binding with B7, promoting NK cell proliferation and increased cytokine secretion (Nandi et al, 1994). Recently, B7-1 was reported to bind to an unidentified receptor on murine NK cells and trigger cytolytic activity (Chambers et al, 1996). Moreover, this signal was able to override the inhibitory signals imparted by MHC class I molecules. Human NK cell lines derived from the YT line also express CD28, and the cytolytic activity of these cell lines is enhanced by the interaction of CD28 with B7 (Azuma et al, 1992*b*; Montel et al, 1995). Moreover, fresh human NK cells have been found to lyse B7-1-expressing tumour cells preferentially (Dessureault and Gallinger, 1996).

Other groups have described varied effects of B7-1 expression on in vivo growth of B16 melanoma. In some cases, B7-1 did not affect the growth pattern (Chen et al, 1994; Townsend et al, 1994), whereas in others it resulted in complete rejection of B16 tumours, a response that was mediated by NK cells and CD8⁻ cells (Wu et al, 1995). These apparently conflicting results probably reflect differing levels of B7-1 expression, as it has been reported that only clones expressing high levels of B7-1 were rejected completely, whereas low-expressing clones grew more slowly but were not rejected (Wu et al, 1995). The results we present here are compatible with this possibility. The clone used in our experiments expressed a comparable level of B7-1 to the low-expressing clone in the latter study. Under in vitro conditions, B7-expressing tumour cells were no more susceptible to lysis by fresh splenocytes than were the parental cells (data not shown). This does not correlate with the in vivo results, but may simply reflect the less than ideal nature of the in vitro assay.

As well as the effects on local tumour growth, it is also of importance to determine the effects of B7 co-stimulatory molecules on systemic immunity. In some studies, B7-expressing tumours have generated protective immunity against a subsequent challenge with parental tumour cells (Cavallo et al, 1995; Gajewski et al, 1996; Dunussi-Joannopoulos et al, 1996) although in other studies such tumours did not elicit any protective immunity (Ramarathinam et al, 1994; Katsanis et al, 1995). Previously, in the moderately immunogenic tumour model (K1735 murine melanoma) we demonstrated that B7-expressing tumours may even reduce the degree of systemic immunity relative to that elicited by the parental tumour (Chong et al, 1996). In contrast, B16 melanoma is a tumour of low intrinsic immunogenicity

 © Cancer Research Campaign 1998

(Dranoff et al. 1993; Vile et al. 1994). The ability of B16 B7-1 cells to elicit systemic protective immunity was examined by inoculation of irradiated tumour cells followed by challenge with live tumour cells. Irradiated B7-1 cells failed to evoke any protection against rechallenge with parental B16 cells, in keeping with reports by other groups (Cavallo et al. 1995; Wu et al. 1995) and consistent with the observation that B7 is generally weak at eliciting systemic immunity against tumours of low intrinsic immunogenicity (Chen et al. 1994).

In contrast, irradiated B16 B7-1 cells consistently protected the majority of mice against challenge with live B16 B7-1 cells. Unlike the local response against B7-expressing tumours, protective immunity was dependent on T cells, as no protection was evident in athymic nude mice. A similar phenomenon was noted in a murine mammary adenocarcinoma model (Zitvogel et al. 1996). Similarly, tumour-infiltrating lymphocytes from a B7-expressing plasmacytoma were able to lyse the B7-expressing tumour cells, but the parental tumour cells were lysed inefficiently (Ramarathinam et al. 1994). It is possible, therefore, that an epitope derived from the B7-1 molecule may act as an antigenic target against which a memory T-cell response is directed. In support of this was the tendency for vaccination with irradiated B16 B7-1 cells to confer a degree of protection against an unrelated syngeneic tumour, CMT93, engineered to express B7-1. Although B7-1 is a 'self' molecule, it may be that in gene-modified tumour cells, where B7-1 is expressed out of its normal context, novel epitopes arise. Alternatively, the requirement for the live challenge tumour cells to express B7-1 may be a reflection of the low degree of protective immunity afforded by inoculation with irradiated B7-1 cells, such that an additional trigger is needed at the time of live challenge.

In this report, we confirm reports by others that the local response against B7-1-expressing B16 melanoma is mediated, at least in part, by NK cells (Wu et al. 1995). We have also shown that the reaction to the B7-2-expressing tumour is similar, and this constitutes the first report describing NK cells as the main effector cells responsible for an in vivo response against a B7-2-expressing tumour. The involvement of NK cells in the local response may help to explain the recent reports concerning the role of host APCs in production of systemic immunity, as elicited by B7-expressing tumour cells (Huang et al. 1996; Cayeux et al. 1997). It is possible that NK cells cause local tumour cell lysis, thus making tumour antigens available in abundance for uptake by professional APCs attracted to the local environment, and these in turn would then present the antigens to T cells efficiently. This situation may be comparable with the systemic immune response associated with tumour cell lysis resulting from activation of a 'suicide gene' such as herpes simplex virus thymidine kinase with ganciclovir treatment (Vile et al. 1997). Therefore, although our results suggest that B7 molecules, by themselves, have limited therapeutic use, it may be useful to attempt co-expression of B7 with a cytokine, such as granulocyte–macrophage colony-stimulating factor (GM-CSF), which promotes maturation of professional APCs. This might then enhance systemic immunity against poorly immunogenic tumours. Experiments to explore such approaches are currently under way.

### ACKNOWLEDGEMENTS

This work was supported by the Imperial Cancer Research Fund. HC held an Imperial Cancer Research Fund Clinical Research Fellowship. We are grateful to the staff of ICRF Biological Resources (Lincolns Inn Fields and Clare Hall) for their expert assistance.

### REFERENCES

Altman DG (1991) *Practical Statistics for Medical Research*. Chapman & Hall: London

Azuma M, Cayabyab M, Buck D, Phillips JH and Lanier LL (1992*a*) CD28 interaction with B7 costimulates primary allogeneic proliferative responses and cytotoxicity mediated by small, resting T lymphocytes. *J Expt Med* **175**: 353–360

Azuma M, Cayabyab M, Buck D, Phillips J and Lanier L (1992*b*) Involvement of CD28 in MHC-unrestricted cytotoxicity mediated by a human natural killer leukemia cell line. *J Immunol* **149**: 1115–1123

Azuma M, Ito D, Yagita H, Okumura K, Phillips JH, Lanier LL and Somoza C (1993) B70 antigen is a second ligand for CTLA-4 and CD28. *Nature* **366**: 76–79

Barlozzari T, Reynolds CW and Herberman RB (1983) In vivo role of natural killer cells: involvement of large granular lymphocytes in the clearance of tumor cells in anti-asialo GM1-treated rats. *J Immunol* **131**: 1024–1027

Bezouska K, Yuen CT, O'Brien J, Childs RA, Chai W, Lawson AM, Drbal K, Fiserova A, Pospisil M and Feizi T (1994) Oligosaccharide ligands for NKR-P1 protein activate NK cells and cytotoxicity. *Nature* **372**: 150–157

Bloom MB, Perry-Lalley D, Robbins PF, Li Y, El-Gamil M, Rosenberg SA and Yang JC (1997) Identification of tyrosinase-related protein 2 as a tumor rejection antigen for the B16 melanoma. *J Immunol* **185**: 453–459

Boon T, Cerottini J-C, Van den Eynde B, van der Bruggen P and Van Pel A (1994) Tumor antigens recognised by lymphocytes. *Annu Rev Immunol* **12**: 337–365

Brunner KT, Engers HD and Cerottini JC (1976). The $^{51}$Cr release assay as used for the quantitative measurement of cell-mediated cytolysis in vitro. In *In Vitro Methods in Cell-mediated and Tumor Immunity*, Bloom BR and David JR (eds) pp 94–106. Academic Press: London

Cavallo F, Martin-Fontecha A, Bellone M, Heltai S, Gatti E, Tornaghi P, Freschi M, Forni G, Dellabona P and Casorati G (1995) Co-expression of B7-1 and ICAM-1 on tumors is required for rejection and the establishment of a memory response. *Eur J Immunol* **25**: 1154–1162

Cayeux S, Richter G, Noffz G, Dorken B and Blankenstein T (1997) Influence of gene-modified (IL-7, IL-4, and B7) tumor cell vaccines on tumor antigen presentation. *J Immunol* **158**: 2834–2841

Chambers BJ, Salcedo M and Ljunggren H-G (1996) Triggering of natural killer cells by the costimulatory molecule CD80 (B7-1). *Immunity* **5**: 311–317

Chen L, Ashe S, Brady WA, Hellstrom I, Hellstrom KE, Ledbetter JA, McGowan P and Linsley P (1992) Costimulation of antitumor immunity by the B7 counterreceptor for the T lymphocyte molecules CD28 and CTLA-4. *Cell* **71**: 1093–1102

Chen L, McGowan P, Ashe S, Johnson J, Li Y, Hellstrom I and Hellstrom K (1994) Tumor immunogenicity determines the effect of B7 costimulation on T cell-mediated tumor immunity. *J Expt Med* **179**: 523–532

Chong H, Hutchinson G, Hart IR and Vile RG (1996) Expression of co-stimulatory molecules by tumor cells decreases tumorigenicity but may also reduce systemic antitumor immunity. *Human Gene Ther* **7**: 1771–1779

Colombo MP and Forni G (1996) Immunotherapy I: cytokine gene transfer strategies. *Cancer Metastasis Rev* **15**: 317–328

Dessureault S and Gallinger S (1996) Allogeneic lymphocyte responses to B7-1 expressing human cancer cell lines. *J Surg Res* **64**: 42–48

Djeu JY, Heinbaugh JA, Holden HT and Herberman RB (1979) Augmentation of mouse natural killer cell activity by interferon and interferon inducers. *J Immunol* **122**: 175–181

Dranoff G, Jaffee E, Lazenby A, Golumbek P, Levitsky H, Brose K, Jackson V, Hamada H, Pardoll D and Mulligan RC (1993) Vaccination with irradiated tumor cells engineered to secrete murine granulocyte-macrophage colony-stimulating factor stimulates potent, specific, and long-lasting anti-tumor immunity. *Proc Natl Acad Sci USA* **90**: 3539–3543

Dunussi-Joannopoulos K, Weinstein HJ, Nickerson PW, Strom TB, Burakoff SJ, Croop JM and Arceci RJ (1996) Irradiated B7-1 transduced primary acute myelogenous leukemia (AML) cells can be used as therapeutic vaccines in murine AML. *Blood* **87**: 2938–2946

Fidler IJ (1970) Metastasis: quantitative analysis of distribution and fate of tumor emboli labeled with $^{125}$I-5-iodo-2'-deoxyuridine. *J Natl Cancer Inst* **45**: 773–782

Franks L and Hemmings V (1978) A cell line from an induced carcinoma of mouse rectum. *J Pathol* **124**: 35–38

© Cancer Research Campaign 1998 

Freeman GJ, Gray GS, Gimmi CD, Lombard DB, Zhou L-J, White M, Fingeroth JD, Gribben JG and Nadler LM (1991) Structure, expression, and T cell costimulatory activity of the murine homologue of the human B lymphocyte activation antigen B7. *J Expt Med* **174**: 625–631

Freeman GJ, Gribben JG, Boussiotis VA, Ng JW, Restivo VAJ, Lombard LA, Gray GS and Nadler LM (1993) Cloning of B7-2: a CTLA-4 counter-receptor that costimulates human T cell proliferation. *Science* **262**: 909–911

Gajewski TF, Fallarino F, Uyttenhove C and Boon T (1996) Tumor rejection requires a CTLA4 ligand provided by the host or expressed on the tumor. *J Immunol* **156**: 2909–2917

Geldhof AB, Raes G, Bakkus M, Devos S, Thielemans K and De Baetselier P (1995) Expression of B7-1 by highly metastatic mouse T lymphomas induces optimal natural killer cell-mediated cytotoxicity. *Cancer Res* **55**: 2730–2733

Gimmi CD, Freeman GJ, Gribben JG, Sugita K, Freedman AS, Morimoto C and Nadler LM (1991) B-cell surface antigen B7 provides a costimulatory signal that induces T cells to proliferate and secrete interleukin 2. *Proc Natl Acad Sci USA* **88**: 6575–6579

Habu S, Fukui H, Shimamura K, Kasai M, Hagai Y, Okumura K and Tamaoki N (1981) In vivo effects of anti-asialo GM1. *J Immunol* **127**: 34–38

Harding F and Allison JP (1993) CD28-B7 interactions allow the induction of CD8+ cytotoxic T lymphocytes in the absence of exogenous help. *J Expt Med* **177**: 1791–1796

Huang A, Bruce A, Pardoll D and Levitsky H (1996) Does B7-1 expression confer antigen-presenting cell capacity to tumors in vivo? *J Expt Med* **183**: 769–776

Huang AYC, Golumbek P, Ahmadzadeh M, Jaffee E, Pardoll D and Levitsky H (1994) Role of bone marrow-derived cells in presenting MHC class I-restricted tumor antigens. *Science* **264**: 961–965

Katsanis E, Xu Z, Bausero MA, Dancisak BB, Gorden KB, Davis G, Gray GS, Orchard PJ and Blazar BR (1995) B7-1 expression decreases tumorigenicity and induces partial systemic immunity to murine neuroblastoma deficient in major histocompatibility complex and costimulatory molecules. *Cancer Gene Ther* **2**: 39–46

Lane P, Gerhard W, Hubele S, Lanzavecchia A and McConnell F (1993) Expression and functional properties of mouse B7/BB1 using a fusion protein between mouse CTLA4 and human gamma 1. *Immunology* **80**: 56–61

Lanier L (1997) Natural killer cells: from no receptors to too many. *Immunity* **6**: 371–378

Linsley PS and Ledbetter JA (1993) The role of the CD28 receptor during T cell responses to antigen. *Annu Rev Immunol* **11**: 191–212

Montel AH, Morse PA and Brahmi Z (1995) Upregulation of B7 molecules by the Epstein–Barr virus enhances susceptibility to lysis by a human NK-like cell line. *Cell Immunol* **160**: 104–114

Naftzger C, Takechi Y, Kohda H, Hara I, Vijayasaradhi S and Houghton AN (1996) Immune response to a differentiation antigen induced by altered antigen: a study of tumor rejection and autoimmunity. *Proc Natl Acad Sci USA* **93**: 14809–14814

Nandi D, Gross J and Allison J (1994) CD28-mediated costimulation is necessary for optimal proliferation of murine NK cells. *J Immunol* **152**: 3361–3369

Pardoll DM (1993) Cancer vaccines. *Immunol Today* **14**: 310–316

Ramarathinam L, Castle M, Wu Y and Liu Y (1994) T cell costimulation by B7/BB1 induces CD8 T cell-dependent tumor rejection: an important role of B7/BB1 in the induction, recruitment, and effector function of antitumor T cells. *J Expt Med* **179**: 1205–1214

Raulet DH (1996) Recognition events that inhibit and activate natural killer cells. *Curr Opin Immunol* **8**: 372–377

Razi-Wolf Z, Freeman GJ, Galvin F, Benacerraf B, Nadler L and Reiser H (1992) Expression and function of the murine B7 antigen, the major costimulatory molecule expressed by peritoneal exudate cells. *Proc Natl Acad Sci USA* **89**: 4210–4214

Riccardi C, Santoni A, Barlozzari T, Puccetti P and Herberman RB (1980) In vivo natural reactivity of mice against tumor cells. *Int J Cancer* **25**: 475–486

Sambrook J, Fritsch EF, Maniatis T (1984) *Molecular Cloning: a Laboratory Manual*. Cold Spring Harbor Laboratory Press, NY

Tepper RI and Mule JJ (1994) Experimental and clinical studies of cytokine gene-modified tumor cells. *Human Gene Ther* **5**: 153–164

Townsend S, Su F, Atherton J and Allison J (1994) Specificity and longevity of antitumor immune responses induced by B7-transfected tumors. *Cancer Res* **54**: 6477–6483

Townsend SE and Allison JP (1993) Tumor rejection after direct costimulation of CD8+ T cells by B7-transfected melanoma cells. *Science* **259**: 368–370

Vile RG and Hart IR (1993) In vitro and in vivo targeting of gene expression to melanoma cells. *Cancer Res* **53**: 962–967

Vile RG, Nelson JA, Castleden S, Chong H and Hart IR (1994) Systemic gene therapy of murine melanoma using tissue specific expression of the *HSVtk* gene involves an immune component. *Cancer Res* **54**: 6228–6234

Vile RG, Chong H and Dorudi S (1996) The immunosurveillance of cancer: specific and non-specific mechanisms. In *Tumor Immunology*, Dalgleish AG and Browning MJ (eds) pp. 7–38. University Press: New York

Vile RG, Castleden S, Marshall J, Camplejohn R, Upton C and Chong H (1997) Generation of an anti-tumour immune response in a non-immunogenic tumour: HSVtk killing in vivo stimulates a mononuclear cell infiltrate and a Th1-like profile of intratumoral cytokine expression. *Int J Cancer* **71**: 267–274

Vujanovic NL, Yasumara S, Hirabayashi H, Lin WC, Watkins S, Herberman RB and Whiteside TL (1995) Antitumor activities of subsets of human IL-2-activated natural killer cells in solid tissues. *J Immunol* **154**: 281–289

Whiteside TL and Herberman RB (1995) The role of natural killer cells in immune surveillance of cancer. *Curr Opin Immunol* **7**: 704–710

Wu T-Z, Huang AYC, Jaffee EM, Levitsky HI and Pardoll DM (1995) A reassessment of the role of B7-1 expression in tumor rejection. *J Expt Med* **182**: 1415–1421

Yeh K-Y, Pulaski BA, Woods ML, McAdam AJ, Gaspari AA, Frelinger JG and Lord EM (1995) B7-1 enhances natural killer cell-mediated cytotoxicity and inhibits tumor growth of a poorly immunogenic murine carcinoma. *Cell Immunol* **165**: 217–224

Zitvogel L, Robbins PD, Storkus WJ, Clarke MR, Maeurer MJ, Campbell RL, Davis CG, Tahara H, Schreiber RD and Lotze MT (1996) Interleukin-12 and B7.1 co-stimulation cooperate in the induction of effective antitumor immunity and therapy of established tumors. *Eur J Immunol* **26**: 1335–1341

© Cancer Research Campaign 1998